# Plant-Derived Antioxidants in Disease Prevention 2018

**DOI:** 10.1155/2018/2068370

**Published:** 2018-12-02

**Authors:** Renata Szymanska, Pavel Pospíšil, Jerzy Kruk

**Affiliations:** ^1^Department of Medical Physics and Biophysics, Faculty of Physics and Applied Computer Science, AGH University of Science and Technology, Reymonta 19, 30-059 Krakow, Poland; ^2^Department of Biophysics, Faculty of Science, Centre of the Region Haná for Biotechnological and Agricultural Research, Palacký University, Šlechtitelů 27, Olomouc 78371, Czech Republic; ^3^Department of Plant Physiology and Biochemistry, Faculty of Biochemistry, Biophysics, and Biotechnology, Jagiellonian University, Gronostajowa 7, 30-387 Krakow, Poland

Plant-derived antioxidants are a large group of natural products with reducing or radical-scavenging capacity. Due to their potent preventive as well as therapeutic actions, these compounds receive a great deal of attention not only from scientists but also from pharmacologists and physicians.

The maintenance of redox homeostasis plays a central role in health and disease prevention. Oxidative stress is generated by an imbalance between reactive oxygen species (ROS) and antioxidants. An excess of ROS leads to the degradation of lipids, proteins, and nucleic acids and thus may lead to the oxidative damage of cells as a consequence of the overexpression of oncogenes, mutagen formation, induction of atherogenic activity, or inflammation. Oxidative stress is suggested to play a major role in the pathogenesis of cardiovascular diseases, neurodegeneration, cancers, immune disorders, diabetes, aging, and others. Plants, especially dietary fruits and vegetables, are a rich source of antioxidants. It is postulated that antioxidants show health benefits through direct reduction of oxidative stress. In the body, an antioxidant network works in concert through several different mechanisms: ROS scavenging, termination of lipid peroxidation, or chelating of metals. Despite the fact that a broad knowledge of antioxidant structures, properties, and biological actions has been gathered, many aspects still require clarification and further studies. Relatively little is known about the cellular mechanisms of their therapeutic potential, interactions with other compounds, appropriate dosage, and effectiveness of treatment (especially their effect in randomized clinical trials). Furthermore, the bioactiveness of a large number of natural compounds remains unknown. Well-known antioxidants, as well those newly discovered, raise hopes for their use in the prevention and treatment of the abovementioned diseases.

In this special annual issue, an attempt has been made to gather articles that update our understanding about the role of plant-derived antioxidants in disease prevention. These reports fill the gaps in the field of antioxidant research, allow better understanding of their action, and facilitate their future usage in disease prevention and treatment. The special issue compiles eleven (11) excellent articles including two (2) reviews and nine (9) research papers, which show current and recent developments in plant-derived antioxidant research.

The review by A. B. Enogieru et al. summarizes the latest knowledge about the neuroprotective mechanisms of plant-derived rutin—a glycoside of the flavonoid quercetin. Rutin has a great potential to be a therapeutic agent in different neurodegenerative diseases. The second interesting review by B. M. Kim describes the therapeutic option of saikosaponins (triterpene saponins) isolated from *Bupleurum* against a variety of age-related disorders.

ROS play a major role in the pathogenesis of numerous diseases. It is believed that the application of exogenous antioxidants is a promising strategy to suppress oxidative stress associated with those disorders or via other still not recognized mechanisms. In this special issue, most of the research articles show that plant-derived compounds (or their extracts) act successfully as suppressors of oxidation and can be used in the future as therapeutic agents. The published papers show either data obtained on cell lines or on the animal models. Articles presented in this annual issue show the antioxidant and protective effects from plant extracts as well as from single compounds.

The research article by I. Rjeibi et al. shows that the methanol extract of *Lycium europaeum* has a protective effect on liver and kidney injuries induced by cisplastin. These authors describe the phytochemical composition of the plant extract, its antioxidant activity, and hepatorenal injury biomarkers.

Likewise, J. M. dos Santos et al. evaluated the chemical profile of the water extract from *Guazuma ulmifolia* stem bark and leaves on different cell lines (erythroleukemia cells, human leucocytes), as well as on model animals. They showed that *Guazuma* extracts can play a role as an adjuvant in doxorubicin chemotherapy. Next, extracts from three Mediterranean food plants (*Carthamus lanatus*, *Cichorium intybus*, and *Cichorium spinosum*) which are a rich source of phenolic compounds were examined by D. Stagos et al.'s group. They have reported that those extracts may prevent diseases associated especially with endothelium damage.

M. Zhu et al. conducted the research on gastrodin—a compound isolated from the medicinal Chinese herb “Tianma.” In this research paper, the authors attempted to explain the cardioprotective mechanisms of gastrodin. They show that the positive effect of gastrodin is connected with the upregulation of 14-3-3n levels. The study of K. Vǎnková et al. showed the antiproliferative and antioxidative effects of chlorophylls (chlorophyll a/b, chlorophyllin, pheophytin a) on pancreatic cancer cell lines. The protective effects of aucubin against myocardial infarction-induced cardiac remodeling was reported by Z. Yang et al. It seems that aucubin acts through the activation of the NOS/NO pathway, which reduces, i.e., ROS production.

J. Peng et al.'s group has evaluated the cataract-preventing function of p-coumaric acid. They demonstrated that p-coumaric acid suppresses H_2_O_2_-induced human lens epithelial cell apoptosis through the MAPK signaling pathway. K. Shanmugam et al. have investigated the cardioprotective effect of fisetin—a natural flavonoid—using a Langendorff isolated heart perfusion system. L. Sun et al. studied the protective effect of the herb hydroxysafflor yellow A during nitrosative stress in neurons. Nitrosative stress in the brain is associated with various neurodegenerative disorders. The results obtained suggest that hydroxysafflor yellow A protects neurons from nitrosative stress.

Taken altogether, the data presented in this special annual issue cover a series of topics addressing the role of plant-derived antioxidants in different oxidative stress-related disease prevention. We believed that the papers published in this special issue not only enrich our understanding of the physiological action of natural products but also provide promising perspectives on their future usage as therapeutic agents. We are sure that all the information provided in this issue will cover a broad range of interests.

